# A novel pathway for amyloids self-assembly in aggregates at nanomolar concentration mediated by the interaction with surfaces

**DOI:** 10.1038/srep45592

**Published:** 2017-03-30

**Authors:** Siddhartha Banerjee, Mohtadin Hashemi, Zhengjian Lv, Sibaprasad Maity, Jean-Christophe Rochet, Yuri L. Lyubchenko

**Affiliations:** 1 Department of Pharmaceutical Sciences, University of Nebraska Medical Center, 986025 Nebraska Medical Center, Omaha, NE 68198-6025, USA; 2 Department of Medicinal Chemistry and Molecular Pharmacology, Purdue University, West Lafayette, Indiana, USA; 3 Purdue Institute for Integrative Neuroscience, Purdue University, West Lafayette, Indiana, USA

## Abstract

A limitation of the amyloid hypothesis in explaining the development of neurodegenerative diseases is that the level of amyloidogenic polypeptide *in vivo* is below the critical concentration required to form the aggregates observed in post-mortem brains. We discovered a novel, on-surface aggregation pathway of amyloidogenic polypeptide that eliminates this long-standing controversy. We applied atomic force microscope (AFM) to demonstrate directly that on-surface aggregation takes place at a concentration at which no aggregation in solution is observed. The experiments were performed with the full-size Aβ protein (Aβ42), a decapeptide Aβ(14-23) and α-synuclein; all three systems demonstrate a dramatic preference of the on-surface aggregation pathway compared to the aggregation in the bulk solution. Time-lapse AFM imaging, in solution, show that over time, oligomers increase in size and number and release in solution, suggesting that assembled aggregates can serve as nuclei for aggregation in bulk solution. Computational modeling performed with the all-atom MD simulations for Aβ(14-23) peptide shows that surface interactions induce conformational transitions of the monomer, which facilitate interactions with another monomer that undergoes conformational changes stabilizing the dimer assembly. Our findings suggest that interactions of amyloidogenic polypeptides with cellular surfaces play a major role in determining disease onset.

Misfolding and aggregation of amyloidogenic proteins is associated with a vast number of neurodegenerative diseases including Alzheimer’s disease (AD), Parkinson’s disease (PD), and Huntington’s disease (HD), termed protein aggregation diseases^1^,^2^. Numerous studies have shown that amyloidogenic proteins are capable of spontaneous assembly into aggregates and eventually form fibrillar structures found in amyloid or amyloid-like deposits^3^,^4^. These studies have led to the amyloid cascade hypothesis, which posits that the disease onset involves a spontaneous assembly of amyloidogenic polypeptide, and that the accumulation of aggregates defines the disease state^5^,^6^,^7^. Numerous studies support this hypothesis that currently is considered the main model for the onset of AD, PD, HD, and other diseases. Although the amyloid cascade hypothesis cannot explain all facts related to the development of these diseases, it remains the major underlying hypothesis of in *vitro* and *in vivo* studies related to the molecular mechanisms of amyloid aggregation causing neurodegenerative diseases^8^. A strong support for the amyloid cascade hypothesis comes from recent studies that demonstrated that antibody-based immunotherapy against Aβ improved cognition in a dose-dependent manner^9^. Evidence of similarities of structural features of aggregates extracted from amyloid plaques with those of Aβ aggregates assembled *in vitro* provide additional support for the use of *in vitro* Aβ aggregation studies for understanding Aβ structural dynamics *in vivo*^10^. Amyloid aggregates can be visualized by electron microscopy and atomic force microscope (AFM) (e.g. refs 11 and 12 and references therein), and structural techniques including X-ray and NMR applied to fibrils have revealed a highly ordered arrangement of protein monomers^13^. However, recent studies have led to the discovery that oligomers rather than fibrils are neurotoxic^3^,^14^,^15^. Studies of these transient oligomeric species have been enabled by the development of novel approaches^16^,^17^ and have shown that at the very early aggregation stage, unstructured monomers form stable dimers due to structural transitions of monomers. In turn, these results suggest that the lack of structure in monomers can facilitate the aggregation. However, there is a serious complication with translating current knowledge on amyloid aggregation *in vitro* to understand the aggregation process *in vivo* —namely, the concentrations of amyloidogenic polypeptides are dramatically different *in vivo* versus *in vitro*. For example, whereas the critical concentration for the spontaneous aggregation of Aβ peptide *in vitro* is in the micromolar range, physiological concentrations of Aβ are in the low nanomolar range^18^,^19^. At such low concentrations, Aβ aggregation cannot occur *in vitro*. Intracellular crowding effects^18^ can facilitate the aggregation process, but these do not solve the problem. Recently, we have found that dimers of α-synuclein (α-Syn) could be assembled at nanomolar concentrations, if the target monomer is tethered to a surface^20^. These data led us to hypothesize that binding to the surface can be a factor dramatically facilitating the aggregation process. This hypothesis is supported by recent studies in which the assembly of large α-Syn aggregates on a glass surface was observed with the protein concentration in the nanomolar range^21^.

In the present study, we developed an approach enabling us to directly test our hypothesis. We utilized AFM to image assembly of aggregates on surfaces and compare this effect with their assembly in the bulk solution. We used full-length Aβ protein (Aβ-42), its aggregation-prone segment Aβ(14-23) peptide and the full-size α-Syn. The experiments demonstrate that all these proteins on mica surface assemble into aggregates at nanomolar concentration with essentially no aggregation propensity in the bulk solution. Time-lapse AFM imaging experiments in solution demonstrate that assembled aggregates can be released to the solution to act as seeds for the aggregation in bulk solution. Computational modeling allowed us to reveal the mechanism of the accelerated on-surface aggregation process. Given that the on-surface aggregates are oligomeric in nature, which are known to be the most neurotoxic species, we hypothesize that prevention of the on-surface aggregation should block the disease-prone process and can be considered a means for the development of future preventions and treatments for Alzheimer’s and similar neurodegenerative protein aggregation diseases.

## Results and Discussion

### Surface aggregation of Aβ-42

**Figure 1:**
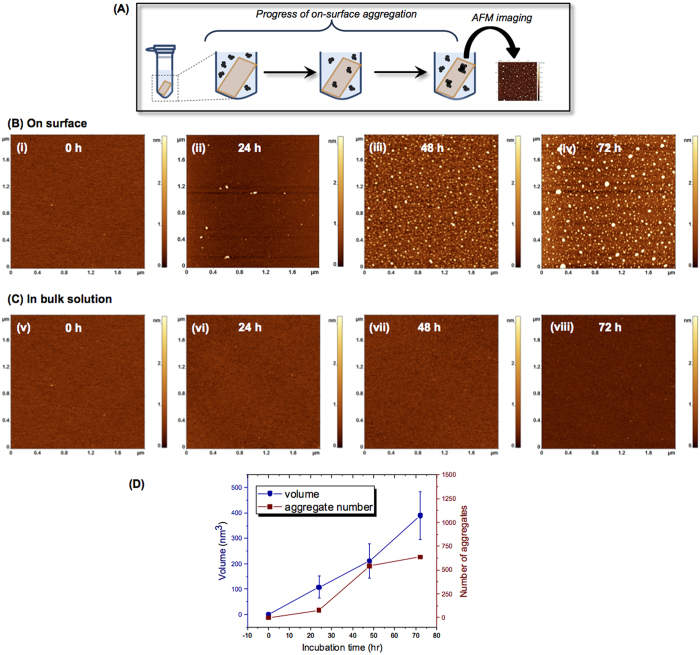
Effect of surface on Aβ-42 (100 nM) aggregation. (**A**) Schematic of the experimental set-up to monitor the on-surface aggregation. APS-functionalized mica surfaces were incubated in 100 nM Aβ-42 solution for different time intervals. Specimens were then taken out from the solution, rinsed, and then imaged using AFM. (**B**) AFM topographic images of the on-surface aggregation at different incubation time (i) 0 h, (ii) 24 h, (iii) 48 h and (iv) 72 h. (**C**) AFM topographs (i–iv) show the aggregation in bulk solution at different time intervals of 0 h, 24 h, 48 h and 72 h, respectively. (**D**) The plot shows the increase in both volume and number of aggregates with time for on-surface aggregation.

To directly test the hypothesis that surface interactions facilitate the self-assembly of amyloidogenic polypeptides, we performed systematic AFM studies of the on-surface aggregation of full size Aβ-42, Aβ(14-23) peptide and α-Syn protein at the nanomolar range. The on-surface aggregation schematically is shown in Fig. 1A. The mica sheets functionalized with 1-(3-aminopropyl) silatrane (APS) were immersed in a 100 nM solution of Aβ-42 in 10 mM sodium phosphate buffer (pH 7.4). After a certain incubation time, a mica sheet was removed from the solution, rinsed with water, dried and imaged with AFM. The samples were prepared for incubation times 0 h, 24 h, 48 h and 72 h, and typical images are shown in Fig. 1B, plates i-iv, respectively. The surface for the initial sample (0 h, plate (i)) is clean, but globular aggregates appear after a 24 h incubation time (plate (ii)), and the surface is quite densely coated with aggregates after 48 h (plate (iii)). The aggregate sizes are larger for the 72 h sample (plate (iv)). In parallel with the on-surface aggregation, aggregation of the same solution of the protein in the absence of a mica surface (aggregation in the bulk solution) was performed. A set of typical images is shown in Fig. 1C. No aggregation was detected for these samples. Only after 72 h of incubation in the bulk solution, a few globular features are noticeable (Fig. 1C, plate viii).

To quantitatively characterize the aggregation process, we measured the aggregate volume and number at all incubation times, and the data are shown in Fig. 1D and Supplementary Fig. 1. The distribution of aggregate volumes is approximated with a single Gaussian for the 24 h incubation with a maximum of 107.2 nm^3^ (Supplementary Fig. 1A), but the distributions are bimodal for two longer aggregation times (48 h (Supplementary Fig. 1B) and 72 h (Supplementary Fig. 1C)), indicating the formation of two sets of aggregates. The sizes of aggregates that appear after a 72 h incubation in the bulk solution were also measured, and the distribution is shown in Supplementary Fig. 1D. The overall number of counts is low due to a low yield of globular aggregates, and their sizes are more than 50 times smaller than those found for the aggregates formed on the surface. Thus, interaction with the surface dramatically enhances the Aβ-42 aggregation process.

Next, to characterize the aggregation process further, we performed time-lapse AFM imaging experiments in which images were taken over the same surface area continuously after injecting 100 nM Aβ-42 protein in 10 mM phosphate buffer (pH 7.4) into the fluid cell. AFM images for selected observation times are shown in Supplementary Fig. 2A. Frame (i) shows the image of the surface prior to adding the protein, illustrating that the surface is clean. The next frame (ii) shows the image taken after 10 min, and here again no protein aggregates are seen. As the incubation time increases, new aggregates appear on the surface. For example, frame (iii) illustrates that quite a large number of aggregates appear after 5 h incubation. Comparison with the images taken after 6 h demonstrates the dynamics of the aggregation process in which aggregates are formed and dissociate and grow. For example, an aggregate circled in yellow in frame (iii) is no longer present in (iv), and the aggregate circled in yellow in (iv) disappears in (v), suggesting that these aggregates have dissociated from the surface. At the same time, a new aggregate circled in green appears in (iv). The aggregate indicated by a dotted black circle remains throughout the time-period of imaging (Supplementary Fig. 2iii–vii) and serves as a marker to monitor the changes occurring in other aggregates. The aggregates marked with a yellow dotted circle dissociate from the surface, whereas the new aggregates which appear on the surface are indicated with a green dotted circle. The results are summarized graphically in Supplementary Fig. 2B. In this graph, the time-dependent changes of overall volume and number of aggregates are plotted. The graphs demonstrate that the particle volumes increase over time monotonically, although the number of aggregates is not monotonic, indicating that aggregation occurs as a dynamic process in which aggregates can dissociate from the surface. To illustrate these dynamics for individual aggregates, we numbered a few particles as shown in Supplementary Fig. 2 iii-vii. For example, the aggregate marked ‘1’ stays in plates iii and iv (Supplementary Fig. 2A) but disappears in plate v. The aggregate marked ‘2’ shows up in plate iv and disappears in plate vii (Supplementary Fig. 2A). Measurement of the aggregate sizes (Supplementary Table 1) demonstrated that some of the aggregates grow in size leading to an increase in volume (aggregates ‘3’, ‘4’, ‘5’ and ‘6’), whereas some dissociate from the surface (aggregates ‘1’ and ‘2’). To estimate the subunit numbers of the oligomers, we compared the volumes determined here with volumes of isolated Aβ-42 oligomers of specific sizes obtained by photoinduced cross-linking^22^. These measurements (data not shown) show that the heptamer and decamer have volumes of 60.3 ± 9.7 nm^3^ and 73.4 ± 12.8 nm^3^ respectively, suggesting that the oligomers formed after 8 h of the time-lapse experiment correspond to the oligomer order of heptamer to decamer.

### Surface aggregation of peptide Aβ(14-23)

**Figure 2:**
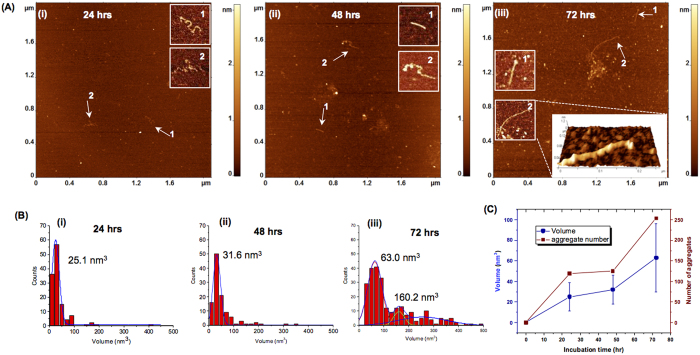
Effect of surface on aggregation of Aβ(14-23). (**A**) AFM images of on-surface aggregation of 100 nM Aβ(14-23) peptide at different time intervals: (i) 24 h, (ii) 48 h, (iii) 72 h show the presence of globular aggregates along with fibrils (highlighted with white arrows). The insets show zoomed images of the fibrillar features and a 3D view of a fibril has been shown as an inset in frame (iii). (**B**) Shows the volume distribution of the globular aggregates for (i) 24 h, (ii) 48 h and (iii) 72 h. (**C**) Plot showing the increase in both volume and number of aggregates with the increase in incubation time.

The stretch of Aβ peptide spanning residues 14 through 23 is the segment of the Aβ-42 peptide (designated as Aβ(14-23) from hereon) that adopts β-sheet structure when Aβ-42 assembles into fibrils, and Aβ(14-23) itself forms fibrils in solution^23^,^24^. The on-surface aggregation experiments were performed under the same conditions as in the above described experiments (100 nM Aβ(14-23), 10 mM sodium phosphate buffer (pH 7.4) at room temperature), and the results are shown in Fig. 2. A few aggregates appear as globular features after 24 h (Fig. 2A-plate i) and their sizes have increased at the 72 h incubation time point (Fig. 2A, plate iii). Fibrillar features were also observed and are indicated with arrows. Zoomed images of the fibrils are shown as insets (Fig. 2Ai–iii), and a 3D view is shown in Fig. 2A(iii). Statistical analysis for the globular aggregates sizes was performed, and the data are shown in Fig. 2B. There is a considerable growth of the aggregates between 48 h (Fig. 2B(ii)) and 72 h (Fig. 2B(iii)), with a bimodal distribution reflecting the presence of large aggregates evident in the histogram corresponding to the later time point (Fig. 2B(iii)). A graph in which these values as well as the number of aggregates were plotted against incubation time (Fig. 2C) illustrates a monotonic growth of aggregates in size and number. As a control, aggregation in the bulk solution was monitored in parallel. Typical AFM images for these samples are shown in Supplementary Fig. 3. Only a few aggregates were formed after 72 h, and no fibrillar features were found.

Time lapse experiments, similar to those carried out with the full-length Aβ42 peptide as described above, were also performed for the Aβ(14-23) peptide. The same surface area was imaged continuously after injecting 100 nM of Aβ(14-23) peptide in 10 mM sodium phosphate buffer in a fluid cell. Supplementary Fig. 4A shows typical images of aggregates at different time intervals: frame (i) 10 minutes after protein addition; (ii) 4 h; (iii) 5 h, and (iv) 6 h respectively. Frame (i) illustrates the presence of a very small number of globular aggregates after 10 min of peptide injection. As time progresses, the number and size of aggregates increases as shown in frames (ii) to (iv). Dynamics of association/dissociation of amyloid aggregates with the surface were also observed in time lapse experiments for this small fragment Aβ(14-23). The black circles in frames (ii), (iii) and (iv) indicate a subset of aggregates and serve as markers to monitor the changes occurring in other aggregates. A few examples of association (green dotted circle) and dissociation (yellow dotted circles) of Aβ(14-23) aggregates are shown in frames (iii) and (iv). The statistics on number and size (volume) of globular aggregates over time are shown in Supplementary Fig. 4B indicating a monotonic increase in both variables with time.

### Surface aggregation of α-synuclein protein

**Figure 3:**
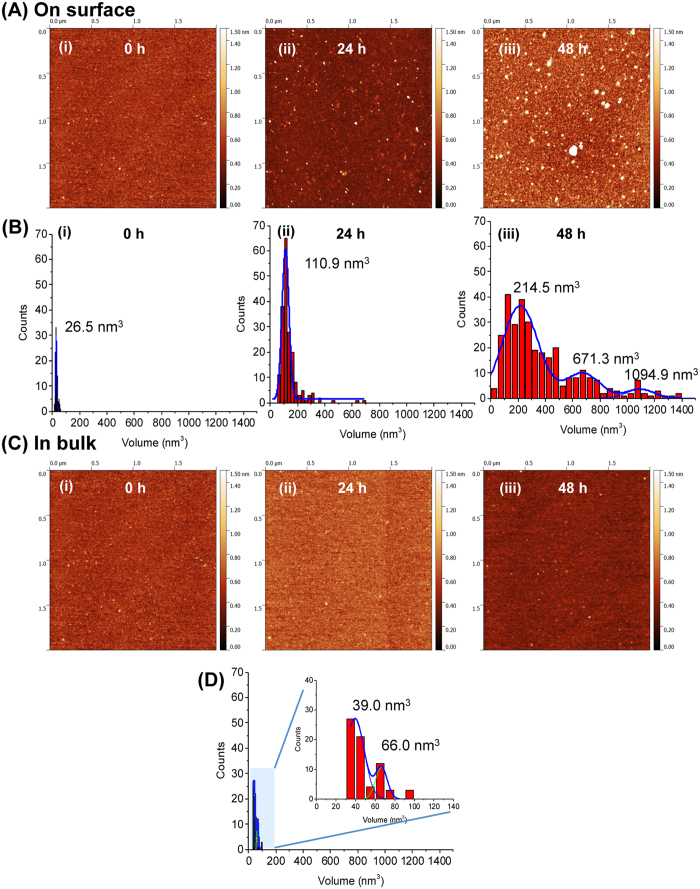
Effect of surface on aggregation of α-Syn (10 nM). **(A)** Shows the AFM topographic images of on-surface aggregation at different time intervals: (i) 0 h, (ii) 24 h, (iii) 48 h. (**B**) Shows the corresponding volume distribution of the aggregates. (**C**) Shows the aggregation of α-Syn in bulk solution. AFM images obtained after depositing an aliquot from the tube at (i) 0 h, (ii) 24 h and (iii) 48 h. (**D**) shows the volume distribution of the aggregates formed in bulk solution after 48 h. The inset represents the zoom scale view where two peaks in the histogram are observed.

To test that the observed enhancement effect of the surface is not limited to Aβ peptides, we performed surface-aggregation experiments with α-synuclein (α-Syn) protein. Given that α-Syn is more than three times larger than Aβ-42 and the fact that we were previously able to image α-Syn monomers^20^, we anticipated being able to visualize the formation of oligomers starting with dimers. The surface-mediated aggregation experiment was carried out similarly to the analyses described above. Similar to the experiments with Aβ peptides, mica sheets were incubated in 10 nM α-Syn solution for different time-periods and imaged with AFM. Typical AFM images for samples corresponding to 0 h, 24 h and 48 h incubation times are shown in Fig. 3A. A few globular features were seen in the initial sample (plate (i)). To characterize their sizes, volume measurements as described in ref. 20 were performed. These measurements are shown in Fig. 3B(i) and they indicate that these features are monomers. After 24 hours (Fig. 3A, plate ii), the particles become brighter and larger in size; the volume measurements (Fig. 3B(ii)) are consistent with the formation of oligomers larger than dimers. Longer incubation (48 hours) leads to the formation of aggregates with a broad range of sizes as evidenced by images in plate (iii) and the corresponding volume distribution (Fig. 3B(iii)). Control experiments for α-Syn aggregation in the bulk solution (Fig. 3C) did not reveal such a pronounced aggregation. Even after a 24 h incubation, the bulk solution did not show aggregates (Fig. 3C, plate ii). The volume measurements for the 48 h sample reveal a major peak at 39 nm^3^ which is very close to the monomer volume (Fig. 3D)^20^. There is a secondary minor peak with a volume value of 66 nm^3^ that can be assigned to dimers-trimers.

Time-lapse measurements were also performed with a 100 nM α-Syn solution. AFM images for 2, 3 and 5 hours of continuous observation, shown in Supplementary Fig. 5, clearly illustrate the time-dependent aggregation of α-Syn. Similar to the previous experiments, the surface was imaged first in 10 mM sodium phosphate buffer (pH 7.4) to confirm that the surface was clean (Supplementary Fig. 5A(i)). Then 100 nM α-Syn was injected onto the surface, and imaging was continued. A considerable number of aggregates were observed after 2 h (Supplementary Fig. 5A(ii)). The number of aggregates was further increased at the 3 and 5 h time points. (Supplementary Fig. 5A(iii, iv)). The volume distribution of the aggregates indicates that the size of the aggregates increases with time (Supplementary Fig. 5B). Similar time-lapse experiments with 10 nM α-Syn demonstrating the surface induced aggregation has also been performed and the data are shown in Supplementary Fig. 6. Apart from globular aggregates, the formation and growth of fibrillar features was also visualized (Supplementary Fig. 6B). In this dataset, an initially small fibrillar complex was observed in plate (i) and found to grow in size over time (plate (ii) and (iii)), but became smaller later on (plate (iv)). This set indicates that the assembled complex is not stable and can dissociate. A control experiment in connection with all of the time-lapse studies mentioned above was performed by imaging a mica surface in 10 mM sodium phosphate buffer (pH 7.4) without any protein for an extended period of time (up to 6 h) (Supplementary Fig. 7). This experiment did not show the appearance of any aggregate like features even after 6 h.

### Computer modeling of the on-surface dimer formation of Aβ(14-23)

**Figure 4:**
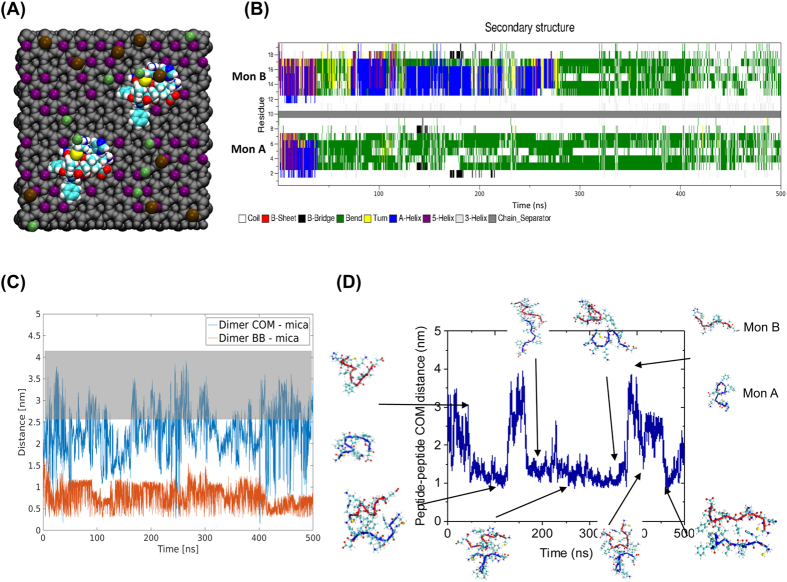
Molecular dynamics simulations of on-surface aggregation of Aβ (14-23) dimers. (**A**) Schematic of the simulation system showing van der Waal representation of the atoms. Grey color is the mica structure excluding potassium cations, K^+^ are purple, Cl^−^ are green, Na^+^ are brown, while peptides are colored using atomic names in VMD. (**B**) Time-dependent change of the secondary structure of the peptides determined using DSSP. Solid gray bar separates the two monomer, with monomer A being below the separator. (**C**) COM distance between dimer and mica surface, blue, and the minimum distance of dimer backbone and mica surface, red, for Mica 1 system as determined by g_mindist. Highlight indicates distance at which dimer is dissociated from mica surface (**D**) The plot shows COM distance between the two Aβ(14-23) peptides in the Mica 1 system; key events of the simulation are highlighted with a cartoon representation of the dimer, blue represents monomer A and red monomer B.

In order to understand the effect of the surface on aggregation, we performed all-atom molecular dynamics (MD) simulations of interactions of Aβ(14-23) monomers with the mica surface, Fig. 4A. Two systems were simulated, Mica1 and Mica2, with initial monomer structure being adopted from^23^; for detailed description of the simulation parameters please see the Methods section.

The interaction of two peptide monomers with each mica surface was simulated, the secondary structure of the monomers is characterized using the defined secondary structure of proteins (DSSP) method^25^ (Fig. 4B). In the Mica1 system, Aβ(14-23) monomer A rapidly interacts with the mica surface; within the first 50 ns of the simulation the monomer approaches the surface. Binding of the monomer is accompanied by its structural transformation, going from having a small helical segment to assuming a bend structure, as seen with the change in secondary structure in Fig. 4B. The interaction between the monomers was characterized by the distance between them as a function of time as shown in Fig. 4C and D. A few snapshots illustrating the peptide structure are indicated along the time trajectory. The surface induces a conformation that is favorable for dimer formation, as is evident from the rapid recruitment of the free monomer and the formation of a dimer bound to the surface, Fig. 4D. Recruitment of the free monomer, monomer B, happens within the first 100 ns of the simulation; the dimerization causes a structural change in the previously free monomer, Fig 4B. However, the newly formed dimer is only formed transiently and for the next ~200 ns forms and dissociates multiple times as is demonstrated by the fluctuation of the dimer-surface plot in Fig 4C. The dimers interact with the surface primarily staying in contact with the surface through interactions involving a few residues and rarely lie fully on the surface. The behavior of the Aβ(14-23) monomers in the Mica2 system is very similar to the Mica1 system, with the exception that the interaction of peptides with the surface is not as strong, and the conformation of the dimers formed is less compact, Supplementary Fig. 8.

### Modeling of Aβ(14-23) dimer formation on DLPE surface

**Figure 5:**
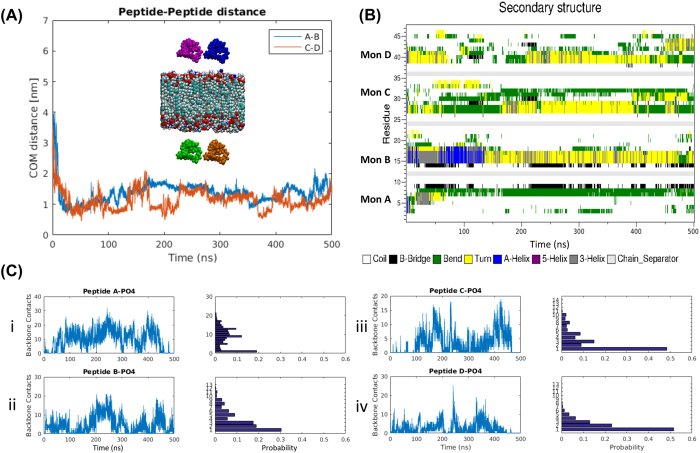
Interaction of four Aβ(14-23) monomers with a DLPE bilayer. (**A**) The distance between two pairs of monomer over time. The inset shows the initial placement of the monomers with respect to the bilayer. Aβ(14-23) monomer A is colored blue, monomer B is colored magenta, monomer C is depicted in brown, monomer D in green, and the bilayer is colored according to the atom names in VMD. Monomers A and B are on the upper leaflet side of the bilayer. (**B**) Shows the secondary structure of individual monomers during the interaction with the bilayer. (**C**) Depicts the frequency of interaction of the four Aβ(14-23) monomers along with the number of backbone contacts between each monomer and the PO_4_ headgroups of the bilayer. The associated frequency histograms are shown to the right.

Next we performed similar MD simulations using the DLPE lipid bilayer system that mimics membrane surfaces^26^,^27^. Two monomers were placed above and below the bilayer (Fig. 5A inset). The initial orientations of the monomers on the outside and inside facing leaflets are opposite of each other to determine if the initial approach orientation has an effect on the surface interaction. Similar to the results for the mica surface, the monomers assemble into dimers, but the process occurs more rapidly. As shown in Fig. 5A, in both dimers, the monomers interact with the surface within 20 ns followed by a rapid dimer formation process. Structural changes occur in monomers upon the interaction with the surface. Fig. 5B demonstrates the structural dynamics of the monomers over the entire simulation time. The initial structure containing small helical segments becomes largely unstructured with β-bridge, turn, or bends. The dimer interacts with the surface and re-organizes on the surface. Through this re-organization, monomers are able to dissociate from and re-associate with the surface – but still remain as dimers. This dynamic behavior is seen as fluctuations in the number and duration of contacts during the simulation, Fig. 5C.

The simulations suggest the following model for the surface effect on dimer formation. Interaction of a monomer with the surface is accompanied by the structural transition of the monomer. Another monomer binds to the immobilized monomer, forms a dimer in which both monomers undergo a structural transition. As a result, the interaction with the surface accelerates the formation of dimers. Compared to our previous simulations for dimer formation by free Aβ(14-23) monomers in which no major structural changes were observed during the first ~200 ns^23^, we have an almost five-fold faster structural transition when the peptides interact with the surface.

## Conclusions

We have shown that the interaction of amyloid proteins with surfaces presents a unique opportunity to allow the protein to assemble into aggregates at concentrations that are non-permissive for aggregation in solution. This finding eliminates a major problem with understanding the spontaneous appearance of plaques in the AD brain due to the apparent gap between known extracellular Aβ concentrations *in vivo* (in the low nM range) and the concentration required for spontaneous aggregation *in vitro* (which is in the μM range). Importantly, the on-surface aggregation is a dynamic process, so the assembled aggregate can dissociate from the surface to the bulk solution. This mechanism is confirmed by direct measurement of the concentration of Aβ-42 aggregates in solution depending on the presence of the mica surface. The data shown in Supplementary Fig. 9 demonstrate that in the presence of mica, the concentration of Aβ-42 aggregates in solution is considerably higher than in the control, and this number grows with time. As a result, these dissociated oligomers can play roles as seeds for aggregation in the bulk solution, or start a neurotoxic effect such as phosphorylation of the tau protein to initiate its misfolding and aggregation followed by neurodegeneration^8^. Additionally, in the vast majority of cases, we found that aggregates formed on the surface are oligomers, which are considered to be the most neurotoxic amyloid aggregates. Although the experimental data presented was for APS-mica, a very similar effect was observed for the bare mica. Additionally, we are currently performing experiments with the use of a lipid bilayer surface. Preliminary results show an even stronger on-surface aggregation effect for the lipid bilayers compared with APS and bare mica. Therefore, we posit that on-surface aggregation is a general mechanism by which neurotoxic amyloid aggregates are produced under physiological conditions.

With regard to applications of this work to AD development, we propose the following model in the framework of the amyloid cascade hypothesis in which interaction of amyloidogenic polypeptides with cellular membranes plays an important role for the disease-related aggregation process. Under normal conditions, the interaction of intracellular or extracellular amyloid proteins with intracellular or extracellular membranes is weak, so small aggregates assemble. These are unstable and dissociate into monomers either on the surface or after dissociation from the membrane. A change in membrane properties leading to an increase in affinity of amyloid proteins to the membrane surface will shift the process to the formation of more stable oligomers that remain intact after dissociation from the surface, and this assembly triggers the disease-related aggregation process. This mechanism does not require elevation of the amyloid peptide concentration, and indeed the concentration of amyloid beta peptide in blood fluctuates weakly regardless of the disease state and does not differ from the controls^28^. Also, the Aβ clearance in late-onset AD patients drops by about one quarter^29^, and only a fraction of the Aβ produced is trapped in amyloid plaques^30^. Our model is in line with recent findings^31^,^32^,^33^,^34^ that demonstrate that the aggregation rate of amyloidogenic proteins measured in the presence of membranes of various types depends on the membrane composition and mechanical properties. Indirect support for the concept of membrane-induced aggregation comes from findings on the elevated yield of Aβ dimers in membrane-containing fractions of blood from AD patients compared with controls^35^. Note as well our direct observation of α-Syn on-surface assembly at nanomolar concentrations when the initial monomer was covalently bound to the surface^20^. Recent NMR studies on the intracellular structure of α-Syn showed that it primarily exists in cells as monomers in an essentially unstructured, compact conformation, but transient interaction of the protein with the membrane was considered^9^. Indeed, the on-surface assembled oligomers comprise a very small fraction of the protein mass in bulk solution, and only a fraction of the aggregates dissociate into the solution, so their detection by NMR is a challenge.

Our surface mediated amyloid aggregation model is a significant departure from the current model in which amyloid aggregation is linked with elevated synthesis of amyloid proteins and their accumulation in the cell to initiate the aggregation process. The lack of evidence for the change of amyloid protein level during the disease progression is a weak point of this model. Our model does not require an elevation of amyloid synthesis as the on-surface assembly can occur at the nanomolar concentration range of the protein. The approaches described in this paper can be extended to various types of surface, so future studies will provide detailed characterization of the on-surface aggregation process and pinpoint possible changes in cellular membrane defining the disease prone state of the organism.

## Methods

### Preparation of protein stock solutions

Aβ-42 protein (AnaSpec, Fremont, CA) and Aβ(14-23) having the sequence HQKLVFFAED (Peptide 2.0 company, VA, USA) were dissolved and sonicated for 5 min in 100 μL of 1,1,1,3,3,3 Hexafluoroisopropanol (HFIP) to destroy pre-aggregated oligomers. Then the solvent was evaporated in vacufuge (1 hr) for complete removal of HFIP. The stock solution of the protein/peptide was prepared in DMSO and kept at −20 °C. Wild-type A140C α-Syn in which the C-terminal alanine was replaced with a cysteine was prepared as described previously^36^. α-Syn solutions were freshly prepared by dissolving 0.4 to 0.8 mg of the lyophilized powder in 200 μL water (the pH has been adjusted to 11 with NaOH), with the addition of 1 μL of 1 M dithiothreitol (DTT) to break disulfide bonds, followed by the addition of 300 μL of 10 mM sodium phosphate buffer (pH 7.0). The obtained solution was filtered through an Amicon filter with a molecular weight cutoff of 3 kDa at 14,000 rpm for 15 min. The filtration was repeated 3 times to completely remove free DTT. The concentration of the stock solutions was determined by spectrophotometry (Nanodrop^®^ ND-1000, DE) using the molar extinction coefficients 1280 cm^−1^·M^−1^ and 120 cm^−1^·M^−1^ for tyrosine and cysteine at 280 nm, respectively. In general, freshly prepared stock solutions were used for all the experiments.

### Sample preparation to monitor the on-surface aggregation

To monitor the effect of surface on aggregation of Aβ42, Aβ(14-23) and α-Syn, 1-(3-aminopropyl) silatrane (APS)-functionalized mica surfaces were used. APS-functionalized mica surfaces were prepared by incubating freshly cleaved mica into 167 μM of APS solution for 30 min, then rinsed with deionized water and dried properly with Argon stream^37^. The small pieces of surfaces were put inside the eppendorf tube (low protein binding tubes) containing 100 nM of protein solution [Aβ42 and Aβ(14-23)] in 10 mM sodium phosphate buffer, pH 7.0. The substrates were taken out at desired time periods and rinsed thoroughly with deionized water and dried under Argon flow for imaging in ambient condition. To compare the on-surface aggregation propensity with that of in bulk solution, a tube containing the same stock of 100 nM protein was incubated along with the tubes with surfaces. 5 μl solution was taken out from this tube and deposited onto the APS-mica at the same time periods to that of surfaces to monitor the aggregation in bulk solution. For α-Syn, a similar method as described above was adopted. The protein concentration used was 10 nM.

### AFM imaging and analysis

AFM images were acquired in tapping mode by Multimode Nanoscope III system (Bruker, Santa Barbara, CA) and MFP-3D AFM (Asylum Research, CA) using TESPA and MSNL (Bruker, CA) probes in air and in buffer medium respectively. The nominal spring constant for TESPA and MSNL probes were ~42 N/m and ~0.1 N/m, respectively. AFM topographic images were analyzed using Femtoscan Online software (Advanced Technologies Center, Moscow, Russia). The volume of the aggregates was obtained from the ‘Enum feature’ and ‘grain analysis’ tool in the software. The data obtained have been plotted in Origin software (OriginPro 2016) to generate the histograms and then they were fitted with Gaussian distribution to measure the mean value and errors^38^.

### Time-lapse imaging of the aggregation event

Time-lapse imaging was carried out in Asylum MFP3D instrument using the MSNL probe (Bruker Corporation). First the functionalized mica surface was imaged in 10 mM sodium phosphate buffer (pH 7.0) to obtain a clean surface, then the desired amount of protein solution was added (100 nM) and imaging was carried out. On-surface aggregation was monitored by scanning the surface at one hour time intervals. The results shown in Supplementary Fig. 6B have been obtained using Nanoscope Multimode VIII system (Bruker, Santa Barbara, CA) in Peakforce mode. A small droplet of 10 nM alpha-synuclein was created under the fluid cell, then the cell was inserted into scanner head and imaging was being continued after proper alignment of the laser. The imaging was continued in the same area to monitor the changes in morphology of the aggregates.

### Molecular dynamics simulations

Molecular dynamics (MD) simulations were conducted using NAMD v 2.10 and employing CHARMM27 force field^39^, extended with INTERFACE FF v1.5 (INTFF) parameters for mica^40^, and the TIP3P water model^41^. A single layer of mica, spanning 52 × 54 Å, was constructed using the INTFF provided structures. Two monomers of Aβ(14-23) were then placed at Center-of-Mass (COM) distance of 2 nm above the mica surface. The initial monomer structure (Figure S9) was adopted from ref. 23. To mimic the experimental design, a Cys residue was added to the N-terminus of the peptide. The index of this Cys residue was set to 0 to keep the context of the other residues as the actual Aβ42 protein. Because we do not know the behavior of the cations on the mica surface, we performed simulations of two different mica surfaces: one, which allowed the K cations to freely move during the simulation, called Mica1, and another system where the K cations were restrained to their crystal positions as obtained from^42^, called Mica2. Both systems were then solvated with TIP3P water molecules. Na^+^ and Cl^−^ ions were then added to neutralize the charges and maintain an ionic concentration of 150 mM. Other details of the simulations setup were adopted from our previous work^23^. 20 ns NVT simulation was then performed. After which 520 ns NPT production simulation, at 1 bar and 300 K, were carried out for each system using Crane at the Holland Computing Center (HCC) and Comet at San Diego Supercomputer Center (SDSC).

Similarly, an equilibrated bilayer containing 128 DLPE (1,2- didodecanoyl-*sn*-glycero-3-phosphoethanolamine) molecules was obtained from http://www.fos.su.se/~sasha/SLipids and used together with Slipids force field parameters^43^ and four Aβ(14-23) monomers, two on each side of the lipid bilayer. The Aβ(14-23) molecules were placed 2 nm COM above the lipid head groups. The rest of the simulation parameters, steps, and duration were the same as the mica simulations.

For analysis, the first 20 ns of the NPT simulation was discarded. The interaction between peptides was examined using the COM distances between each of the peptides. Likewise, the minimum distance between the peptide and the mica layer was also calculated using the COM of each peptide and the Si atoms of the mica surface, this was done using *g_distance*. Similarly for the DLPE system, the distances were calculated with respect to the PO4 groups. Additionally, the backbone interactions of each of the monomers were also monitored using *g_mindist*. To follow the frequency of interaction of each of the peptides as they interact with the surface, the number of contacts between peptide backbone and the surface were monitored; contact being defined as distances less than 1 nm.

## Additional Information

**How to cite this article**: Banerjee, S. *et al*. A novel pathway for amyloids self-assembly in aggregates at nanomolar concentration mediated by the interaction with surfaces. *Sci. Rep.* **7**, 45592; doi: 10.1038/srep45592 (2017).

**Publisher's note:** Springer Nature remains neutral with regard to jurisdictional claims in published maps and institutional affiliations.

## Supplementary Material

Supplementary Information

## References

[b1] Dobson C. M. Principles of protein folding, misfolding and aggregation. Semin Cell Dev Biol 15, 3–16, doi: 10.1016/j.semcdb.2003.12.008 (2004). 15036202

[b2] Ross C. A. & Poirier M. A. Protein aggregation and neurodegenerative disease. Nat Med 10 Suppl, S10–17, doi: 10.1038/nm1066 (2004). 15272267

[b3] Ahmed M. . Structural conversion of neurotoxic amyloid-beta(1-42) oligomers to fibrils. Nat Struct Mol Biol 17, 561–567, doi: 10.1038/nsmb.1799 (2010). 20383142 PMC2922021

[b4] Friedrich R. P. . Mechanism of amyloid plaque formation suggests an intracellular basis of Aβ pathogenicity. Proceedings of the National Academy of Sciences 107, 1942–1947, doi: 10.1073/pnas.0904532106 (2010). PMC2836607 20133839

[b5] Hardy J. An ‘anatomical cascade hypothesis’ for Alzheimer’s disease. Trends Neurosci 15, 200–201 (1992). 1378661 10.1016/0166-2236(92)90033-5

[b6] Hardy J. A. & Higgins G. A. Alzheimer’s disease: the amyloid cascade hypothesis. Science 256, 184–185 (1992). 1566067 10.1126/science.1566067

[b7] Karran E., Mercken M. & De Strooper B. The amyloid cascade hypothesis for Alzheimer’s disease: an appraisal for the development of therapeutics. Nature reviews. Drug discovery 10, 698–712, doi: 10.1038/nrd3505 (2011). 21852788

[b8] Musiek E. S. & Holtzman D. M. Three dimensions of the amyloid hypothesis: time, space and ‘wingmen’. Nat Neurosci 18, 800–806, doi: 10.1038/nn.4018 (2015). 26007213 PMC4445458

[b9] Sevigny J. . The antibody aducanumab reduces Abeta plaques in Alzheimer’s disease. Nature 537, 50–56, doi: 10.1038/nature19323 (2016). 27582220

[b10] Miller L. M. . Synchrotron-based infrared and X-ray imaging shows focalized accumulation of Cu and Zn co-localized with beta-amyloid deposits in Alzheimer’s disease. J Struct Biol 155, 30–37, doi: 10.1016/j.jsb.2005.09.004 (2006). 16325427

[b11] Rambaran R. N. & Serpell L. C. Amyloid fibrils: abnormal protein assembly. Prion 2, 112–117 (2008). 19158505 10.4161/pri.2.3.7488 PMC2634529

[b12] Lyubchenko Y. & Shlyakhtenko L. In Handbook of Clinical Nanomedicine Pan Stanford Series on Nanomedicine 589–616 (Pan Stanford, 2016).

[b13] Tycko R. Physical and structural basis for polymorphism in amyloid fibrils. Protein Sci 23, 1528–1539, doi: 10.1002/pro.2544 (2014). 25179159 PMC4241104

[b14] Benilova I., Karran E. & De Strooper B. The toxic Abeta oligomer and Alzheimer’s disease: an emperor in need of clothes. Nat Neurosci 15, 349–357, doi: 10.1038/nn.3028 (2012). 22286176

[b15] Zhao L. N., Long H., Mu Y. & Chew L. Y. The toxicity of amyloid beta oligomers. Int J Mol Sci 13, 7303–7327, doi: 10.3390/ijms13067303 (2012). 22837695 PMC3397527

[b16] Lyubchenko Y. L. Amyloid misfolding, aggregation, and the early onset of protein deposition diseases: insights from AFM experiments and computational analyses. AIMS Molecular Science 2, 190–210, doi: 10.3934/molsci.2015.3.190 (2015). 27830177 PMC5098429

[b17] Lyubchenko Y. L., Zhang Y., Krasnoslobodtsev A & Rochet J. C. In Handbook of Clinical Nanomedicine: From Bench to Bedside (ed Bawa R., Audette G. & Rubinstein I.) Ch. 16, 978-981-4669-4621-4664 (eBook). Appended to the application. (Pan Stanford Publishing, 2015).

[b18] Hu X. . Amyloid seeds formed by cellular uptake, concentration, and aggregation of the amyloid-beta peptide. Proc Natl Acad Sci USA 106, 20324–20329, doi: 10.1073/pnas.0911281106 (2009). 19910533 PMC2787156

[b19] Grimmer T. . Beta amyloid in Alzheimer’s disease: increased deposition in brain is reflected in reduced concentration in cerebrospinal fluid. Biol Psychiatry 65, 927–934, doi: 10.1016/j.biopsych.2009.01.027 (2009). 19268916 PMC2700302

[b20] Lv Z. . Direct Detection of alpha-Synuclein Dimerization Dynamics: Single-Molecule Fluorescence Analysis. Biophys J 108, 2038–2047, doi: 10.1016/j.bpj.2015.03.010 (2015). 25902443 PMC4407253

[b21] Rabe M. . On-surface aggregation of alpha-synuclein at nanomolar concentrations results in two distinct growth mechanisms. ACS Chem Neurosci 4, 408–417, doi: 10.1021/cn3001312 (2013). 23509977 PMC3605820

[b22] Yamin G., Huynh T. P. & Teplow D. B. Design and Characterization of Chemically Stabilized Abeta42 Oligomers. Biochemistry 54, 5315–5321, doi: 10.1021/acs.biochem.5b00318 (2015). 26241378 PMC5104494

[b23] Lovas S., Zhang Y., Yu J. & Lyubchenko Y. L. Molecular mechanism of misfolding and aggregation of Abeta(13-23). J Phys Chem B 117, 6175–6186, doi: 10.1021/jp402938p (2013). 23642026 PMC3695694

[b24] Zhang Y. & Lyubchenko Y. L. The structure of misfolded amyloidogenic dimers: computational analysis of force spectroscopy data. Biophysical journal 107, 2903–2910, doi: 10.1016/j.bpj.2014.10.053 (2014). 25517155 PMC4269799

[b25] Kabsch W. & Sander C. Dictionary of protein secondary structure: Pattern recognition of hydrogen-bonded and geometrical features. Biopolymers 22, 2577–2637, doi: 10.1002/bip.360221211 (1983). 6667333

[b26] Kucerka N. . Molecular structures of fluid phosphatidylethanolamine bilayers obtained from simulation-to-experiment comparisons and experimental scattering density profiles. J Phys Chem B 119, 1947–1956, doi: 10.1021/jp511159q (2015). 25436970

[b27] Jacoby G. . Metastability in lipid based particles exhibits temporally deterministic and controllable behavior. Scientific reports 5, 9481, doi: 10.1038/srep09481 (2015). 25820650 PMC4377625

[b28] Toledo J. B., Shaw L. M. & Trojanowski J. Q. Plasma amyloid beta measurements - a desired but elusive Alzheimer’s disease biomarker. Alzheimers Res Ther 5, 8, doi: 10.1186/alzrt162 (2013). 23470128 PMC3706955

[b29] Potter R. . Increased *in vivo* amyloid-beta42 production, exchange, and loss in presenilin mutation carriers. Science translational medicine 5, 189ra177, doi: 10.1126/scitranslmed.3005615 (2013). PMC3838868 23761040

[b30] Keaney J. . Autoregulated paracellular clearance of amyloid-beta across the blood-brain barrier. Science advances 1, e1500472, doi: 10.1126/sciadv.1500472 (2015). 26491725 PMC4610013

[b31] Matsuzaki K. How do membranes initiate Alzheimer’s Disease? Formation of toxic amyloid fibrils by the amyloid beta-protein on ganglioside clusters. Acc Chem Res 47, 2397–2404, doi: 10.1021/ar500127z (2014). 25029558

[b32] Galvagnion C. . Chemical properties of lipids strongly affect the kinetics of the membrane-induced aggregation of alpha-synuclein. Proc Natl Acad Sci USA 113, 7065–7070, doi: 10.1073/pnas.1601899113 (2016). 27298346 PMC4932957

[b33] Grey M. . Acceleration of alpha-synuclein aggregation by exosomes. J Biol Chem 290, 2969–2982, doi: 10.1074/jbc.M114.585703 (2015). 25425650 PMC4317028

[b34] Vácha R., Linse S. & Lund M. Surface Effects on Aggregation Kinetics of Amyloidogenic Peptides. Journal of the American Chemical Society 136, 11776–11782, doi: 10.1021/ja505502e (2014). 25068615

[b35] Villemagne V. L. . Blood-borne amyloid-beta dimer correlates with clinical markers of Alzheimer’s disease. J Neurosci 30, 6315–6322, doi: 10.1523/jneurosci.5180-09.2010 (2010). 20445057 PMC6632738

[b36] Krasnoslobodtsev A. V. . Effect of spermidine on misfolding and interactions of alpha-synuclein. PloS one 7, e38099, doi: 10.1371/journal.pone.0038099 (2012). 22662273 PMC3360652

[b37] Lyubchenko Y. L. & Shlyakhtenko L. S. AFM for analysis of structure and dynamics of DNA and protein-DNA complexes. Methods (San Diego, Calif.) 47, 206–213, doi: 10.1016/j.ymeth.2008.09.002 (2009). PMC2667448 18835446

[b38] Serio T. R. . Nucleated conformational conversion and the replication of conformational information by a prion determinant. Science 289, 1317–1321 (2000). 10958771 10.1126/science.289.5483.1317

[b39] Mackerell A. D. Jr., Feig M. & Brooks C. L. 3rd. Extending the treatment of backbone energetics in protein force fields: limitations of gas-phase quantum mechanics in reproducing protein conformational distributions in molecular dynamics simulations. Journal of computational chemistry 25, 1400–1415, doi: 10.1002/jcc.20065 (2004). 15185334

[b40] Heinz H. & Suter U. W. Surface structure of organoclays. Angewandte Chemie (International ed. in English) 43, 2239–2243, doi: 10.1002/anie.200352747 (2004). 15108132

[b41] Jorgensen W. L., Chandrasekhar J., Madura J. D., Impey R. W. & Klein M. L. Comparison of simple potential functions for simulating liquid water. The Journal of Chemical Physics 79, 926–935, doi: 10.1063/1.445869 (1983).

[b42] Heinz H., Lin T. J., Mishra R. K. & Emami F. S. Thermodynamically consistent force fields for the assembly of inorganic, organic, and biological nanostructures: the INTERFACE force field. Langmuir 29, 1754–1765, doi: 10.1021/la3038846 (2013). 23276161

[b43] Jämbeck J. P. M. & Lyubartsev A. P. An Extension and Further Validation of an All-Atomistic Force Field for Biological Membranes. Journal of Chemical Theory and Computation 8, 2938–2948, doi: 10.1021/ct300342n (2012). 26592132

