# Supplementary material for: A novel pathway for amyloids self-assembly in aggregates at nanomolar concentration mediated by the interaction with surfaces
Source: Sci Rep. 2017 Mar 30;7:45592. doi: 10.1038/srep45592 (PMC5372363; doi:10.1038/srep45592)
Supplement: Supplementary Information [file srep45592-s1.pdf]

Supplementary Information

A novel pathway for amyloids self-assembly in aggregates at nanomolar concentration mediated by the interaction with surfaces

Siddhartha Banerjee[1†], Mohtadin Hashemi[1†], Zhengjian Lv[1†], Sibaprasad Maity[1†], Jean-Christophe Rochet[2], and Yuri L. Lyubchenko[1*].
[1]Department of Pharmaceutical Sciences, University of Nebraska Medical Center, 986025 Nebraska Medical Center, Omaha, NE 68198-6025
[2]Department of Medicinal Chemistry and Molecular Pharmacology, Purdue University, West Lafayette, Indiana, USA
[†]These authors contributed equally to the work.

*Correspondence to: ylyubchenko@unmc.edu

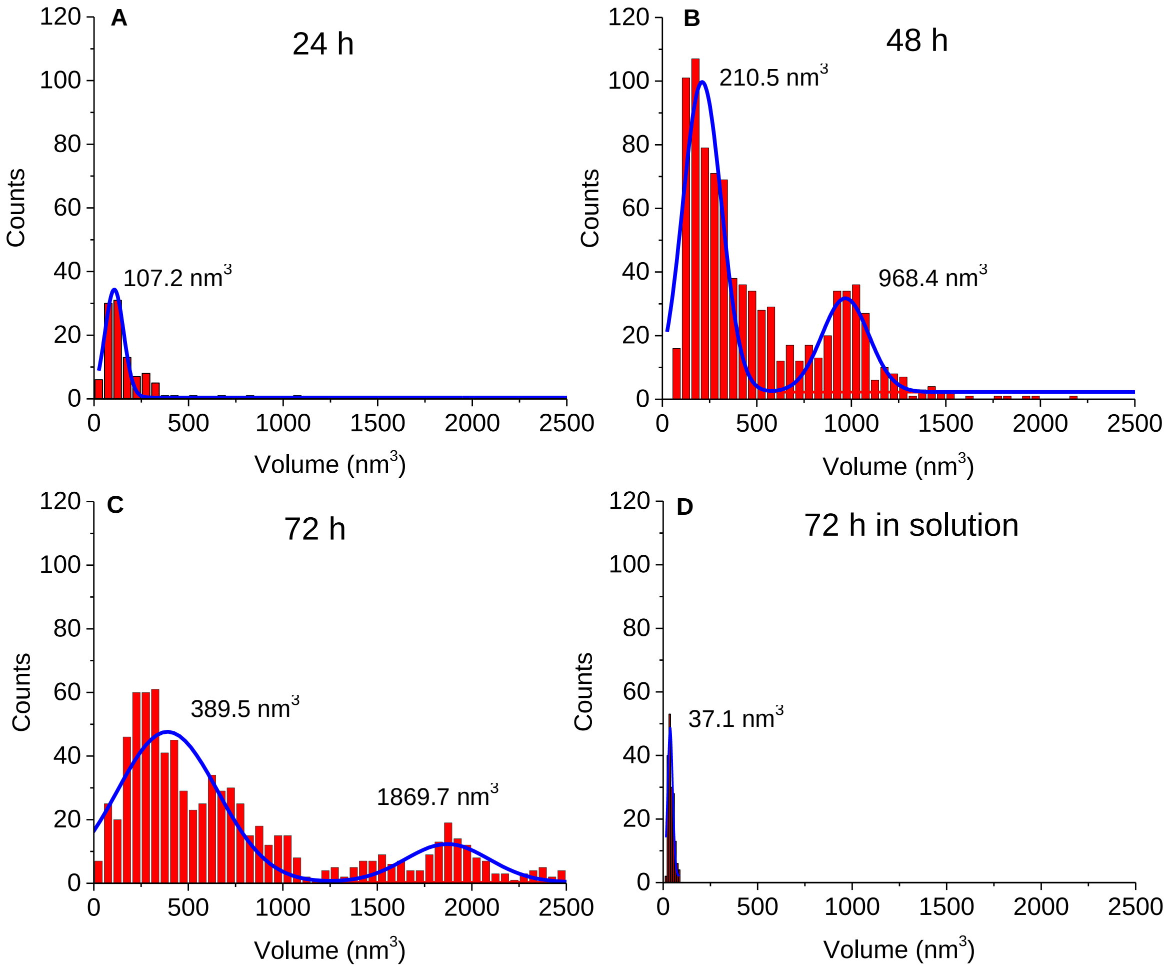

Supplementary Figure 1.

Volume distribution of Aβ-42 aggregates produced due to on-surface aggregation. Volume of the aggregates after (A) 24 h, (B) 48 h and (C) 72 h of incubation. The distribution for 24 h remain unimodal whereas, 48 h and 72 h distribution are bimodal. (D) Shows the volume distribution of aggregates formed after 72 h in the bulk (control experiments). The histograms are fitted with Gaussian distribution. The peak values are indicated in each plot.

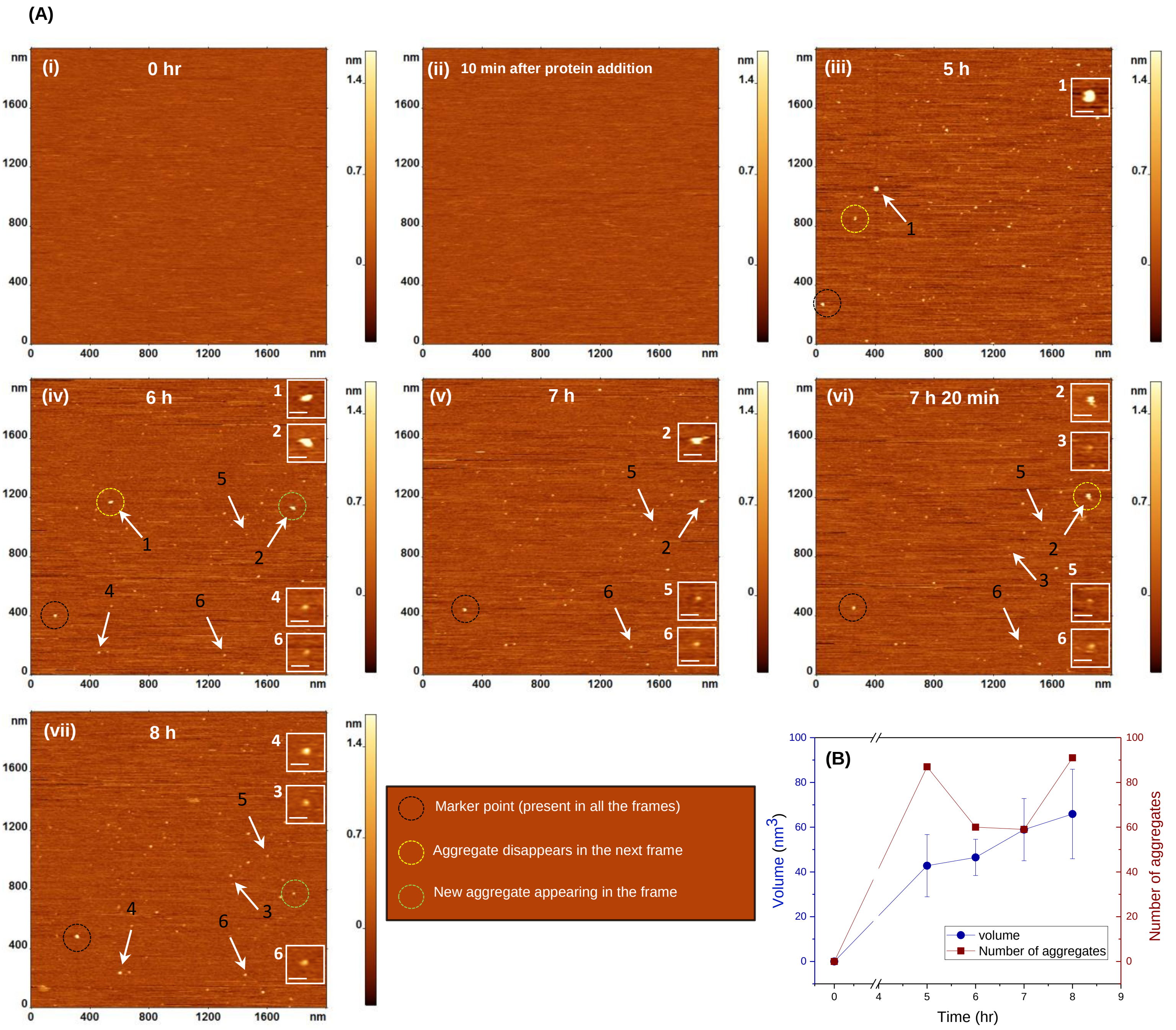

Supplementary Figure 2.

Time-lapse imaging of on-surface aggregation of Aβ-42. (**A**) AFM topographs from (i) to (vii) show the same area of the surface with the Aβ-42 aggregates. The time at which the topographs were recorded indicated on each frame. The marker, disappearance and appearance of the new aggregates, are marked with dotted black, yellow and green circles respectively. Few representative features are marked in the topographs by numbers. The numbers represent the position of that particular aggregate throughout the time-lapse imaging. The insets show the enlarged view of few aggregates (Scale bars: 50 nm). (**B**) The plot shows the changes in volume and number of the aggregates throughout the experimental time period. Monotonous increase in the average volume of the aggregates has been observed, whereas the number of aggregates fluctuates indicating the dynamic behavior of the aggregates on the surface.

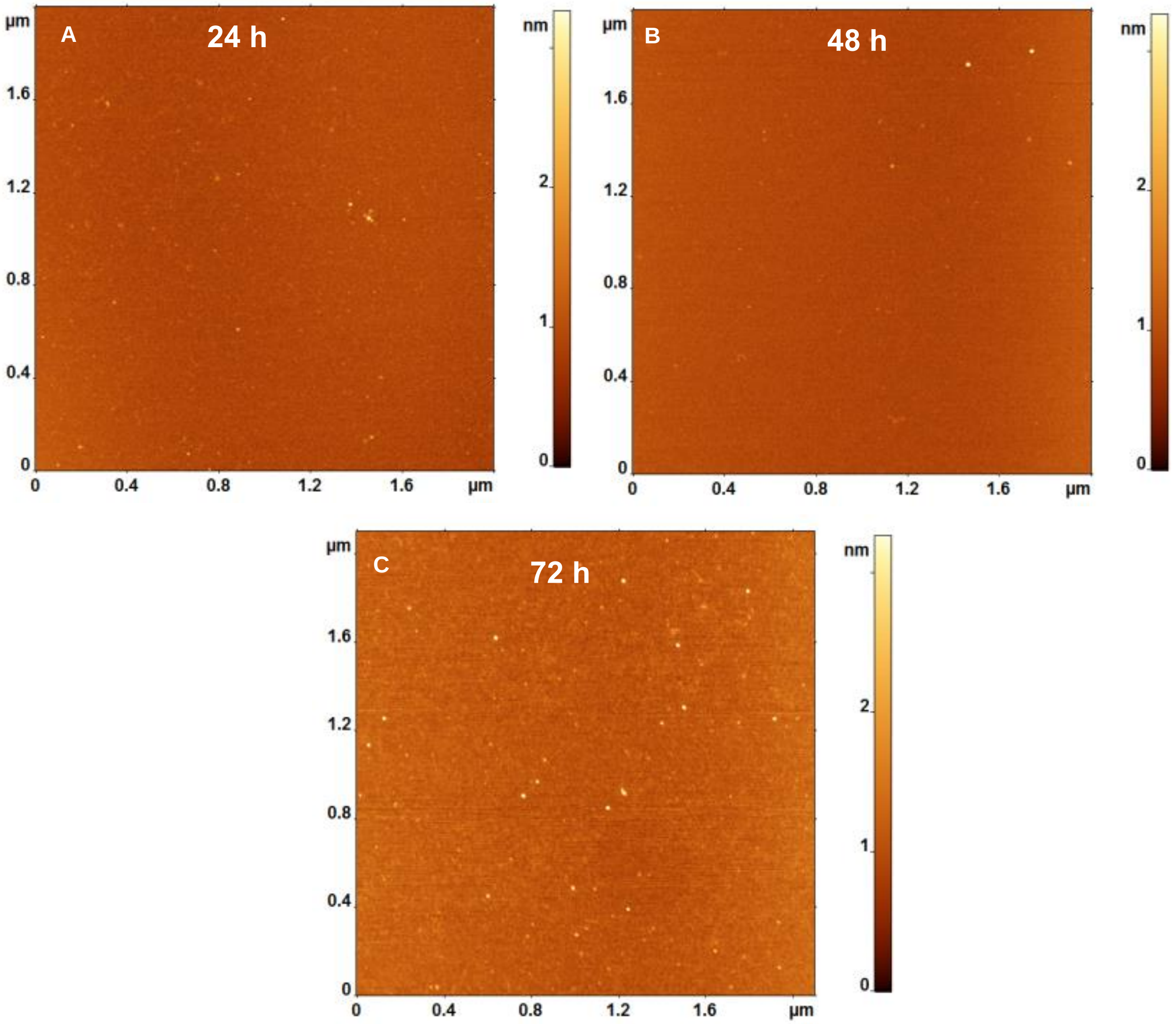

Supplementary Figure 3.

Monitoring the aggregation of Aβ(14-23) in bulk. 100 nM of Aβ(14-23) was incubated in a test-tube and an aliquot was deposited onto APS-mica surface after (A) 24 h, (B) 48 h and (C) 72 h. No fibrillar aggregates were observed. Only few globular features are visible.

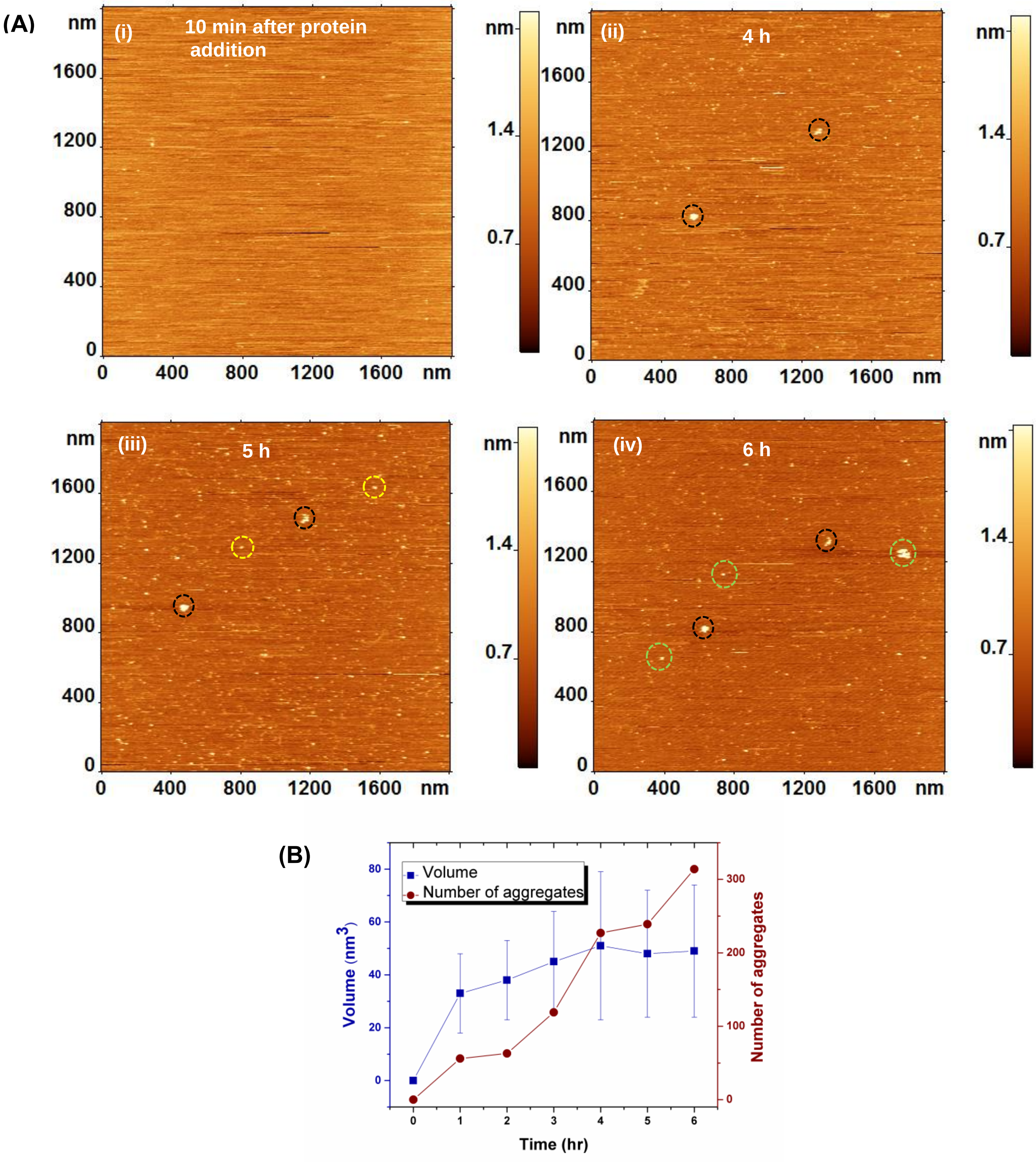

Supplementary Figure 4.

Time-lapse imaging of on-surface aggregation of Aβ (14-23). (**A**) AFM topographic images from (i) to (iv) showing the same area with Aβ(14-23) aggregates at different time intervals indicated in each frame. Aggregates start appearing from the 4 h time point and have increased in number and size at 5 h and 6 h. A few representative aggregates are marked with dotted circles where black, yellow and green circles indicate the marker, disappearance and appearance of the new aggregates, respectively. (**B**) The plot shows the changes in volume and number of the aggregates throughout the experimental time period.

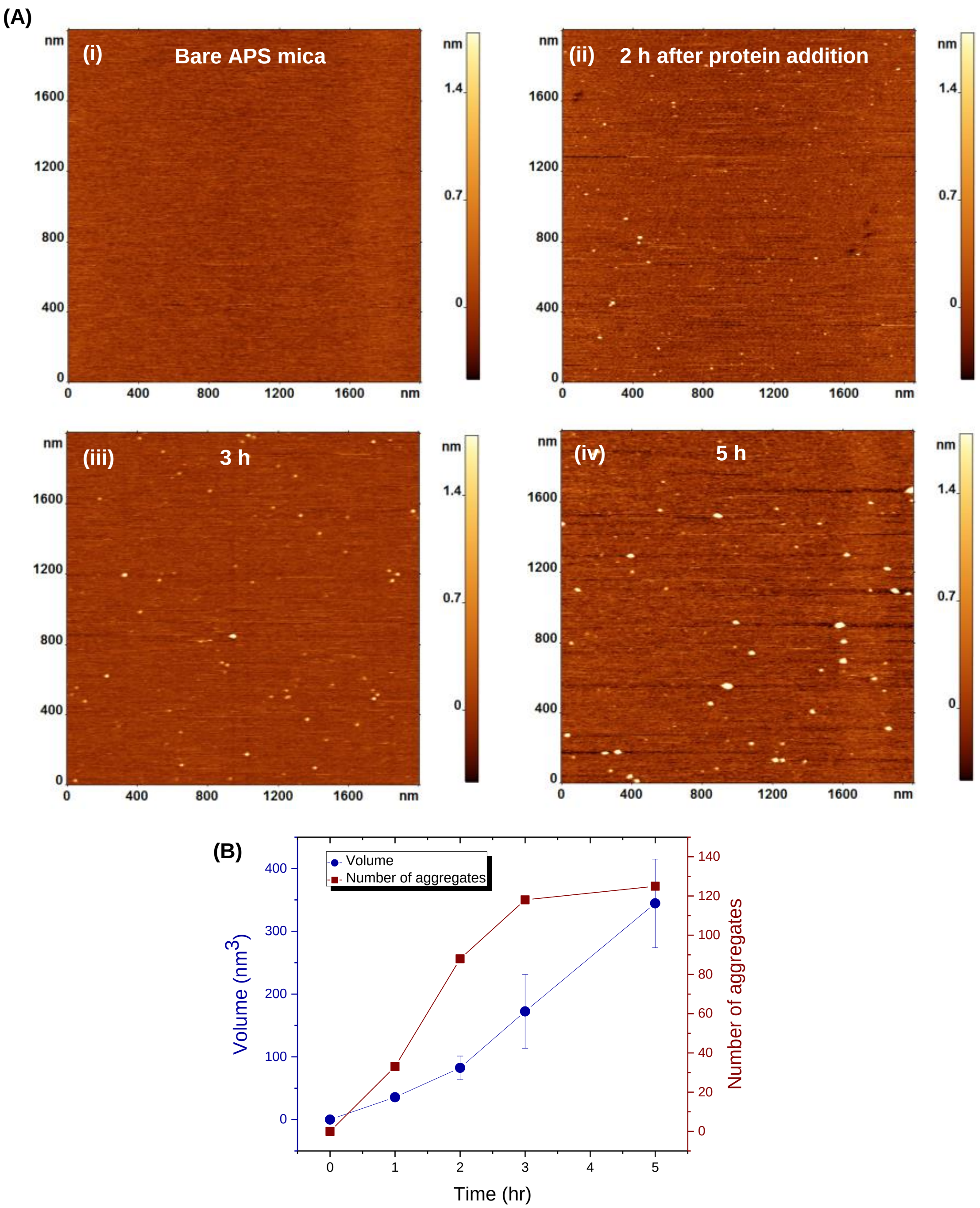

Supplementary Figure 5.

Time-lapse imaging of on-surface aggregation of α-Syn (100 nM). (**A**) AFM topographs showing *in situ* progression of on surface α-syn aggregation at different time intervals (i) before addition of protein, (ii) 2 h, (iii) 3 h and (iv) 5 h after protein addition. Initial clean image of the surface shows that the surface is suitable for time-lapse experiment. Aggregates start appearing after 2 h and they increase in number at 3 h and 5 h indicating the progress of on-surface aggregation. (**B**) The plot summarizes the data for volume and number of aggregates with the time. Both the number and the volume show a monotonic increase.

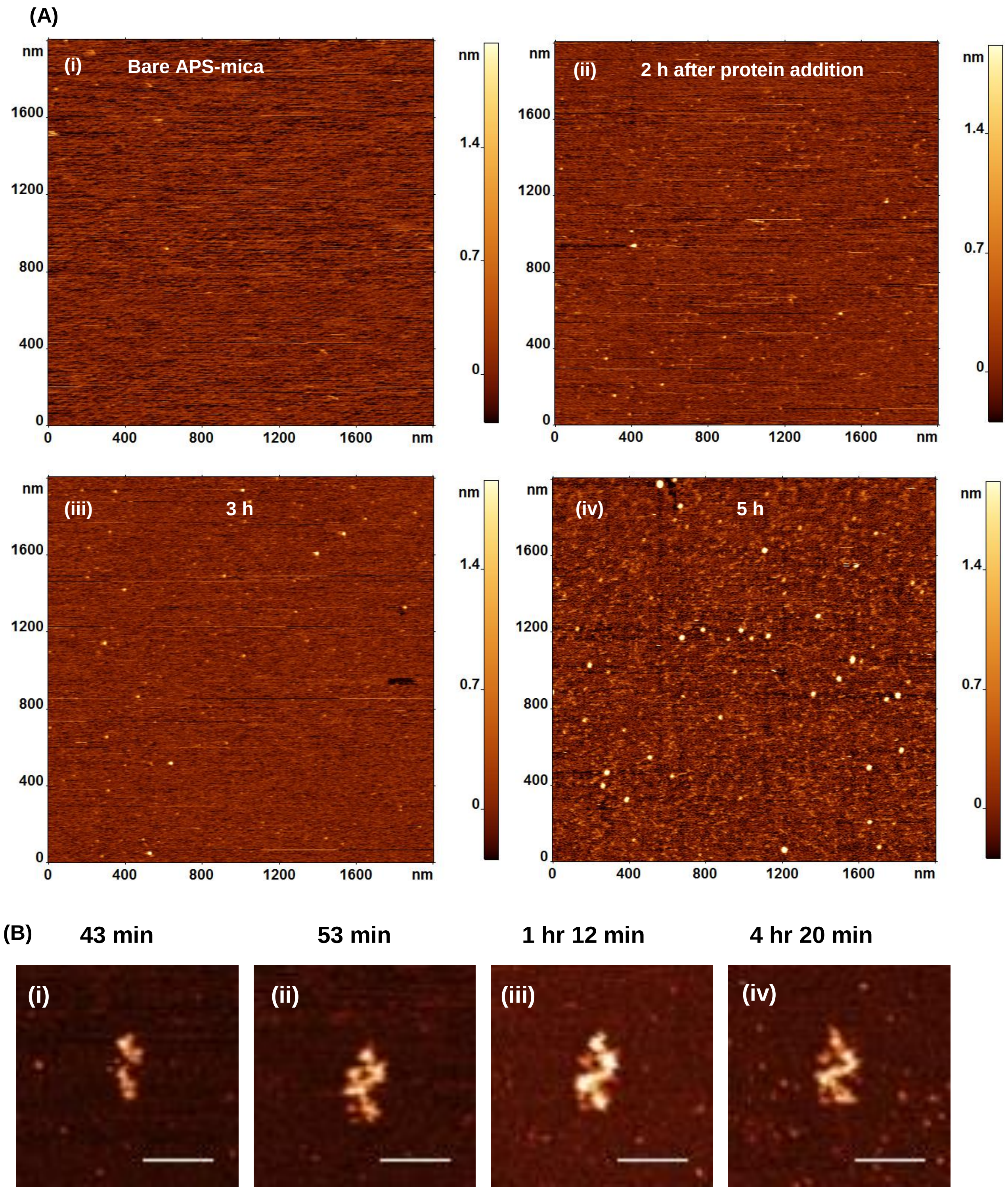

Supplementary Figure 6.

Time-lapse imaging of on-surface aggregation of α-Syn (10 nM). (A) Experimental condition has been kept similar to the time-lapse imaging as described in Fig. S5, except the α-syn concentration. AFM topographs showing *in situ* progression of on surface α-Syn aggregation at different time intervals (i) before addition of protein, (ii) 2 h, (iii) 3 h and (iv) 5 h after protein addition. Initially the surface has been imaged before protein addition to ensure clean surface for time-lapse experiment. Aggregates start appearing after 2 h and they increase in number and size at 3 h and 5 h indicating the progress of on-surface aggregation. (**B**) shows the growth of protofibril like features on an APS-functionalized mica surface. The value above each frame indicates the time point at which the image was acquired. Scale bar: 100 nm and the Z scale: 0-7.0 nm.

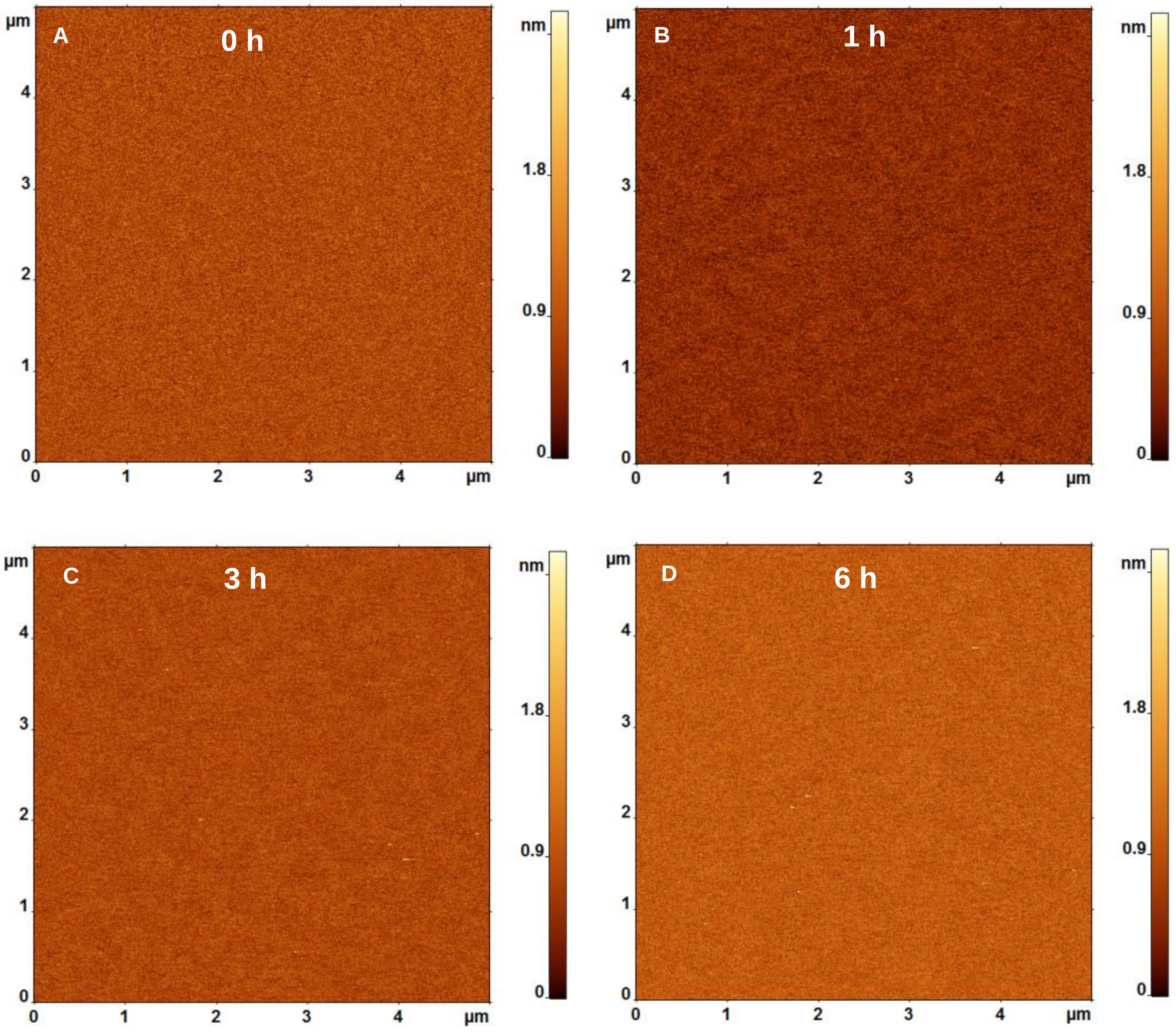

Supplementary Figure 7.

Control experiment for time-lapse measurements. APS-mica surface was imaged under 10 mM sodium phosphate buffer (pH 7.0) at (A) 0 h, (B) 1 h, (C) 3 h and (D) 6 h. The surface does not reveal any aggregate-like features.

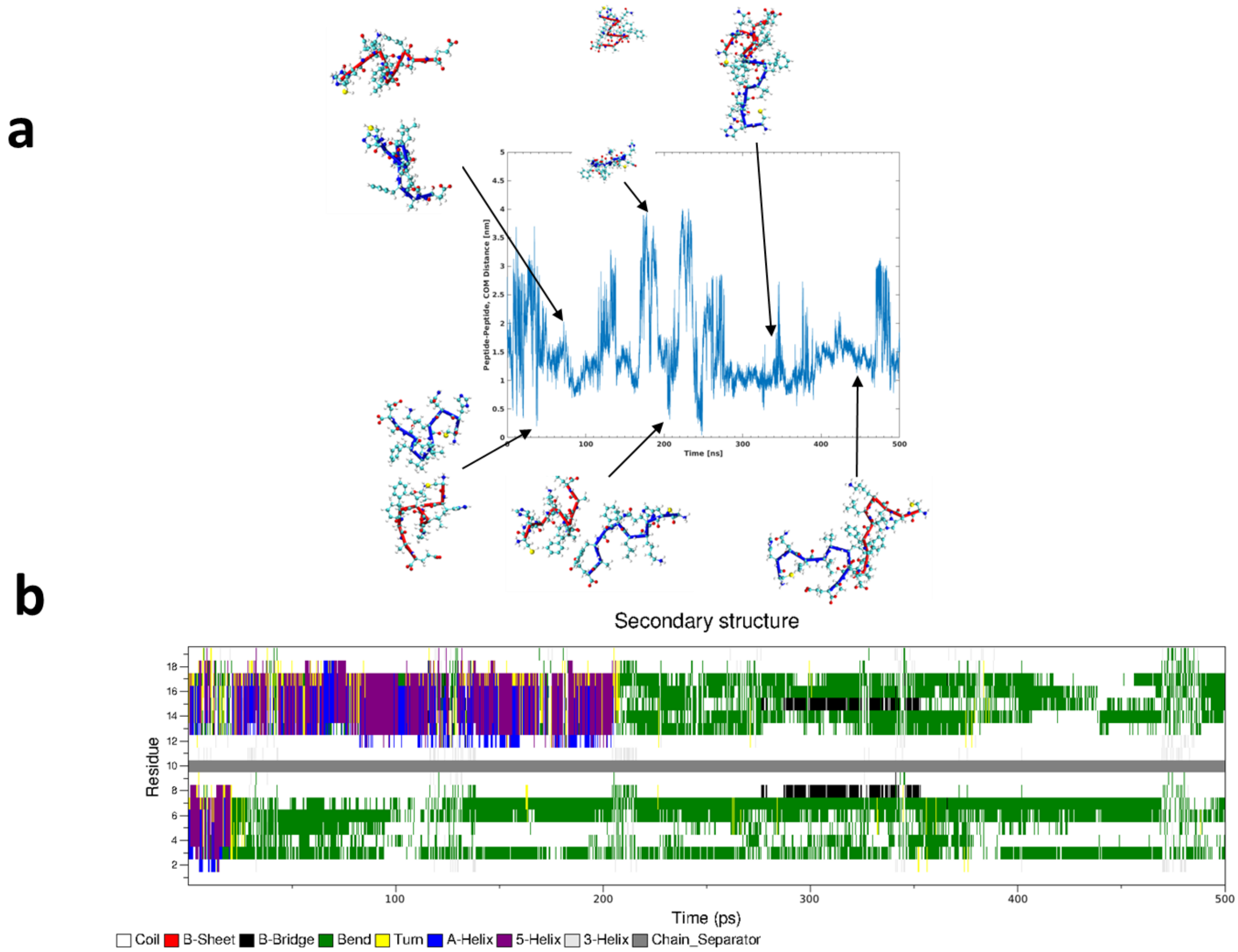

Supplementary Figure 8.
Interactions of Aβ (14-23) monomers and Mica 2, with K+ atoms are fixed to initial positions. (A) COM distance between the two Aβ (14-23) peptides; key events of the simulation are highlighted with a cartoon representation of the dimer, blue represents monomer A and red Monomer B. (B) Time-dependent change of the secondary structure of the peptides. Solid gray bar separates the two monomer, with Monomer A being below the separator.

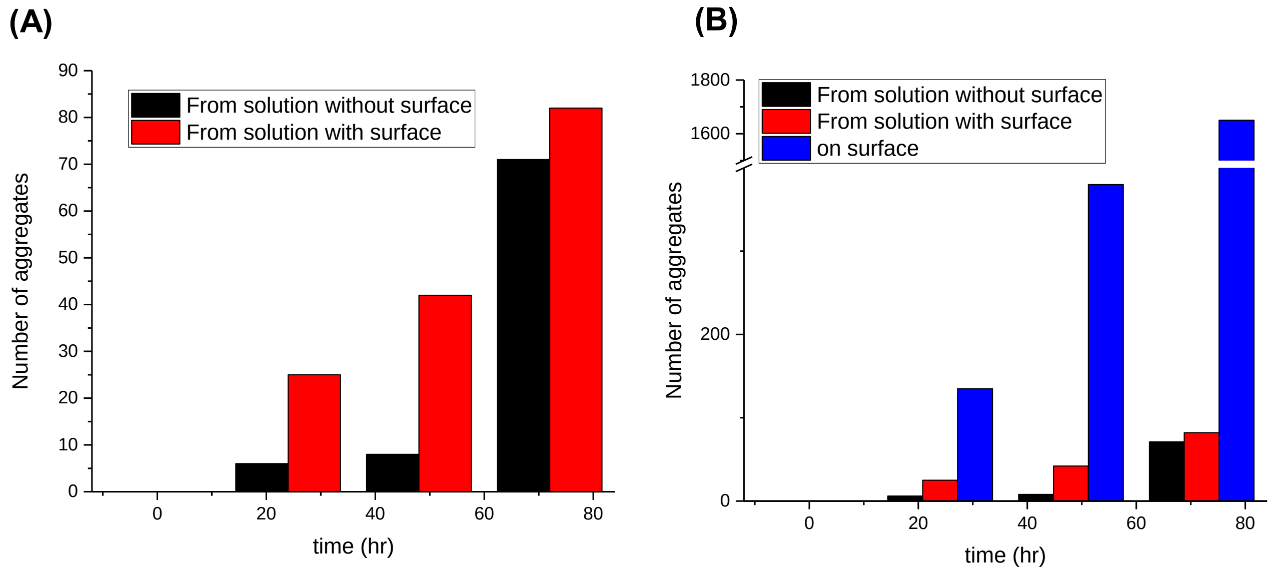

Supplementary Figure 9.

The effect of surface on the accumulation of Aβ-42 aggregates in solution.

The time dependence of the Aβ-42 aggregates concentration in the bulk depending on the presence of mica sheets in the test tubes. Two sets of test tubes were used in parallel with the same Aβ-42 solution. One set contained mica sheet. 10 µl of the protein solution was taken out form the tubes at different time periods of 0 h, 24 h, 48 h and 72 h and analyzed with AFM. (A) The number of aggregates counted for the tubes containing mica sheets (red) versus the control (black). The diagram demonstrates a considerable increase in number of aggregates solutions containing mica. (B) The on-surface aggregation data (blue bars) are added to the diagram in (B).

Supplementary Table 1.

Height and volume analysis of time-lapse AFM imaging of Aβ-42 aggregation shown in Fig 2 and Fig. S2. Feature number represents the particular aggregate shown in Fig. S2.

**Feature**	**Time**	**Height (nm)**	**Volume (nm^3)**
1	5 h	4.9	1031.5
	6 h	3.6	707.3
2	6 h	4.0	925.3
	7 h	3.8	800.2
	7 h 20 mins	3.1	738.8
3	7 h 20 mins	1.0	30.5
	8 h	1.8	62.2
4	6 h	1.3	131.2
	8 h	2.3	252.2
5	6 h	1.0	45.2
	7 h	1.2	60.1
	7 h 20 mins	1.0	50.5
	8 h	1.4	95.8
6	6 h	1.2	90.2
	7 h	1.5	115.2
	7 h 20 mins	1.5	120.2
	8 h	1.8	170.5
